# Current In Vitro and In Vivo Models to Study MCPyV-Associated MCC

**DOI:** 10.3390/v14102204

**Published:** 2022-10-07

**Authors:** Amanda S. W. Loke, Paul F. Lambert, Megan E. Spurgeon

**Affiliations:** McArdle Laboratory for Cancer Research, Department of Oncology, School of Medicine & Public Health, University of Wisconsin, Madison, WI 53705, USA

**Keywords:** Merkel cell polyomavirus, Merkel cell carcinoma, viral oncogenesis, DNA tumor virus, human polyomavirus, preclinical models, murine models, organotypic rafts

## Abstract

Merkel cell polyomavirus (MCPyV) is the only human polyomavirus currently known to cause human cancer. MCPyV is believed to be an etiological factor in at least 80% of cases of the rare but aggressive skin malignancy Merkel cell carcinoma (MCC). In these MCPyV+ MCC tumors, clonal integration of the viral genome results in the continued expression of two viral proteins: the viral small T antigen (ST) and a truncated form of the viral large T antigen. The oncogenic potential of MCPyV and the functional properties of the viral T antigens that contribute to neoplasia are becoming increasingly well-characterized with the recent development of model systems that recapitulate the biology of MCPyV+ MCC. In this review, we summarize our understanding of MCPyV and its role in MCC, followed by the current state of both in vitro and in vivo model systems used to study MCPyV and its contribution to carcinogenesis. We also highlight the remaining challenges within the field and the major considerations related to the ongoing development of in vitro and in vivo models of MCPyV+ MCC.

## 1. Introduction

The etiological role of viruses in human malignancies has been well-established since the discovery of the first identified human tumor virus, Epstein–Barr virus (EBV), in Burkitt’s lymphoma cells in 1964 [1]. Since then, other human tumor viruses have been discovered, namely hepatitis B virus (HBV), hepatitis C virus (HCV), human papillomavirus (HPV), human T cell lymphotropic virus (HTLV-1), Kaposi’s sarcoma-associated herpesvirus (KSHV), and most recently, Merkel cell polyomavirus (MCPyV) (reviewed in [2]). Together, these viruses are estimated to cause at least 15% of human cancers worldwide [3,4]. Our collective understanding of the pathogenesis and oncogenic potential of human tumor viruses has been catalyzed by and dependent upon the development of model systems that mimic the biology of virus-induced diseases. In many cases, the species specificity of human tumor viruses complicates their study and introduces significant challenges in the design of model systems. Nevertheless, preclinical models facilitate the study of several aspects of human tumor virology, including viral entry, the roles of viral and host factors and environmental co-factors in the establishment of persistent infections that are a prerequisite for later development of cancers, viral pathogenesis, and ultimately oncogenesis. Importantly, these model systems also serve as much-needed platforms for translational research into potential prophylactic and therapeutic strategies.

The study of tumor viruses using model systems has contributed significantly to our understanding of what are now recognized as basic tenets of cell and molecular biology, such as RNA transcription, splicing, and polyadenylation [5,6,7], DNA replication [8,9], and nuclear localization [10], to name a few (reviewed in [11,12,13,14]). The establishment of several key principles in cancer biology is also owed in part to the study of tumor viruses, including the discovery of cancer-driving ‘oncogenes’ identified through studies of the avian onco-retrovirus Rous Sarcoma Virus that encodes the *SRC* oncogene [15,16]. The study of double-stranded DNA viruses known as DNA tumor viruses more specifically led to the discovery and functional understanding of another important class of cancer-relevant cellular genes called ‘tumor suppressors’. Many DNA tumor viruses encode proteins that inhibit the function of these cellular gene products that normally suppress cancer, thereby creating conditions conducive to cancer development. For instance, the most commonly disrupted tumor suppressor in human cancers, the cellular p53 gene, was discovered through studies of SV40, a simian polyomavirus that can cause tumors in rodents and transform cells in tissue culture [17,18]. Another family of DNA tumor viruses, the high-risk human papillomaviruses, which cause 5% of human cancers [4,19], also encode proteins that disrupt the function of p53 [20,21]. Another major tumor suppressor, the retinoblastoma protein (pRb), was also discovered through the study of DNA tumor viruses (reviewed in [22]). As many DNA tumor viruses often employ parallel mechanisms to cause cancer, MCPyV proteins also target p53 and pRb protein functions in ways believed to be involved in MCC development [23,24,25].

Among human tumor viruses, MCPyV is the only polyomavirus currently known to cause human cancer, specifically the aggressive cutaneous cancer called Merkel cell carcinoma (MCC). Substantial effort has been made to generate relevant biological models to help further our understanding of MCPyV viral mechanisms and their causal role in the development of MCC. Despite several challenges, including a lack of clarity about MCPyV tropism and the MCC cell of origin, substantial progress in understanding MCPyV’s role in carcinogenesis has been made since the discovery of the virus in 2008 [26]. In this review, we discuss current in vitro and in vivo model systems used to study MCPyV infection and MCPyV+ MCC, highlighting the challenges faced along the path to their development and future considerations for models of this virus-induced disease.

To provide sufficient context for the challenges and considerations of developing preclinical model systems of MCPyV+ MCC, Section 1.1 *(Merkel Cell Carcinoma*), Section 1.2 *(Merkel Cell Polyomavirus),* and Section 2 (*Role of MCPyV in MCC*) outline the discovery of MCPyV virus and its biology, as well as our current understanding of its role in MCC development. Readers more familiar with the topic may choose to proceed directly to Section 3 (*Considerations for designing MCPyV+ MCC Models*).

### 1.1. Merkel Cell Carcinoma (MCC)

Merkel cell carcinoma (MCC) is a cutaneous malignancy with neuroendocrine traits that frequently metastasizes to draining lymph nodes and distant organs [27,28]. MCC was first identified in 1972 [29] and was originally described as “trabecular carcinoma of the skin”. The tumors were observed in patients of advanced age and presented in the dermis or in the adjacent subcutaneous layer. Upon the observation that MCC cells contained neurosecretory granules, a common feature shared by tumors originating from the neural crest and a trait also present in normal Merkel cells [30], the tumor classification was changed to MCC [31,32]. Both neuroendocrine markers, such as chromogranin A, synaptophysin, neural cell adhesion molecule (NCAM), insulinoma-associated 1 (INSM1) and REST Corepressor 2 (RCOR2) [33,34,35,36], and epithelial markers, such as cytokeratins (K) K8, K18, K19 and K20, are detected in MCCs (reviewed in [37,38,39]).

A mnemonic known as ‘AEIOU’ summarizes clinical and epidemiological characteristics in a majority (90%) of MCCs: Asymptomatic/lack of tenderness, Expanding rapidly, Immune suppression, Older than 50 years, and UV-exposed/fair skin [40]. MCC most frequently presents as a benign-appearing growth in older, fair-skinned adults with a history of high sun exposure. MCC risk is also increased in severely immunocompromised individuals such as HIV/AIDS patients [41] or as a result of immunosuppressive therapies associated with the treatment of autoimmune diseases, solid organ transplantation, and cancers [42] (reviewed in [43]).

MCC is a rare yet aggressive cancer. There are approximately 2000 cases of MCC diagnosed per year in the US, and this incidence is expected to increase in the coming years [44,45]. The estimated 5-year overall survival rate of MCC is 51% for local disease, 35% for nodal involvement, and 14% for metastatic MCC [46]. For both local and nodal MCC, surgery and chemoradiation intervention are used as treatment; however, for more advanced or recurrent cases of MCC, systemic therapy involving cytotoxic chemotherapy regimens is required. Recently, independent clinical trials with immune checkpoint blockade-based immunotherapies utilizing avelumab and pembrolizumab that target programmed cell death 1 by PD-1 and its ligand, PD-L1, resulted in clinical response in approximately 30–60% of advanced MCC cases based on RECIST criteria for partial or complete response [47,48]. Both compounds have now been approved for advanced MCC in the United States and European Union [47,48] (reviewed in [49]). With the predicted increase in MCC incidence [44,45], it is imperative to develop more effective therapeutic strategies. As such, the need is critical for in vitro and in vivo model systems that simulate the disease and can act as platforms to develop and test these strategies.

### 1.2. Merkel Cell Polyomavirus (MCPyV)

#### 1.2.1. MCPyV Discovery

Certain risk factors for MCC, specifically immunodeficiency, immunosuppression, and advanced age, are associated with increased susceptibility to viral infection, prompting Feng and colleagues to ask whether MCC had a viral etiology. Digital transcriptome subtraction analysis revealed the presence of non-human transcripts with high homology to genomic sequences of known polyomaviruses in patient-derived MCC tissue samples, leading to their discovery of Merkel cell polyomavirus (MCPyV, also abbreviated as MCV) [26], so-named for its discovery within MCC tumors. It is now recognized that at least 80% of MCC tumors are MCPyV-positive (MCPyV+ MCC), as indicated by the presence of clonal, integrated sequences of the MCPyV viral genome in the host genome [26,50]. Serology studies indicate that most individuals acquire MCPyV at a young age [51] and that the virus is ubiquitous in the general population [52,53,54,55,56], suggesting that MCPyV+ MCC is a rare consequence of virus infection.

#### 1.2.2. Viral Genome Organization and Viral Gene Products

**Figure 1:**
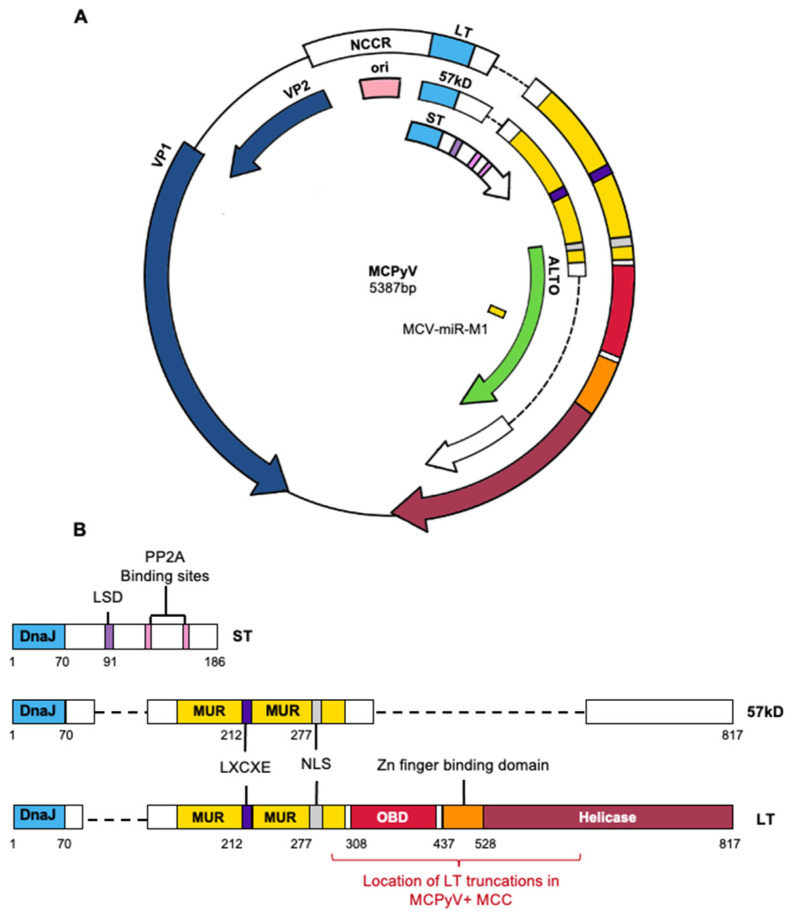
MCPyV genome and T antigens. (**A**) Circularized MCPyV viral genome depicting NCCR, early genes (ST, 57kD, LT, ALTO), late genes (VP1, VP2) and the microRNA miR-m1. Abbreviations: ST—small tumor antigen, 57kD—57 kD tumor antigen, LT—large tumor antigen, ALTO—alternative large tumor open reading frame, NCCR—non-coding control region, ori—origin of replication, VP—viral protein. (**B**) Functional domains of MCPyV T antigens, including DnaJ (light blue), LXCXE (indigo), and Zn finger binding domain (orange). Numbers indicate amino acid position as described in Wendzicki et al. [71]. Truncating mutation locations are indicated by red bar. Abbreviations: LSD (purple)—LT stabilizing domain, MUR (yellow)—MCPyV unique region, NLS (silver)—nuclear localization signal, OBD (red)—origin binding domain.

Similar to other polyomaviruses, MCPyV is a non-enveloped virus that contains a double-stranded, circular DNA genome of 5.4 kilobases (Figure 1A). The viral genome is divided into early and late regions that are separated by the non-coding control region (NCCR), which contains the origin of DNA replication and bi-directional promoters that drive transcription of the early and late mRNAs off of opposite DNA strands. The early region encodes viral proteins involved in replication and amplification of the viral DNA genome that are referred to as tumor antigens (T antigens) based on early studies that determined analogous viral proteins in SV40 and mouse polyomavirus were expressed in tumors arising in rodent species and facilitated tumorigenesis, in part, through the ability of LT to bind and inactivate the tumor suppressors, p53 and pRb [17,18]. The late region encodes structural proteins that form the proteinaceous capsid of the virus, which encapsidates the viral genome to give rise to progeny viruses, and this facilitates virus binding to and entry into cells.

The MCPyV early region encodes three T antigens: large T antigen (LT), small T antigen (ST), and a 57 kD T antigen (57 kD) [26,49] (Figure 1B). The MCPyV LT protein contains many features shared with other polyomavirus LTs, including an LXCXE motif for binding the pRB tumor suppressor protein, an origin-binding domain (OBD), a nuclear localization signal (NLS), and a helicase domain involved in viral DNA replication [57] (Figure 1B). The small T (ST) protein is translated from a single translational open reading frame, part of which is present in the first exon of both LT and 57 kD T protein (Figure 1B). Downstream portions of this ST-encoding ORF that are not shared with LT or 57 kD T encode for a protein phosphatase 2A (PP2A) binding site that is a common feature of STs encoded by other polyomaviruses [58,59]. While ST does not possess any functional domains typically involved in DNA replication, multiple studies have indicated that ST is required for optimal viral genome replication [60,61]. ST contains an LT-Stabilization Domain (LSD) that stabilizes LT expression and prevents its degradation, resulting in enhanced viral replication [62]. The 57 kD T antigen shares the same amino terminus with LT and ST and also contains the MUR, LXCXE motif, and the C-terminal 100 amino acids that are present in LT (Figure 1B) and has been described as an analog of the 17 kD antigen of the SV40 virus [63]. However, there has yet to be definitive evidence for the involvement of 57 kD T antigen in either the viral life cycle or MCPyV-induced tumorigenesis and difficulties expressing this protein in vitro have complicated efforts to fully characterize its functions [64]. The early region also encodes a viral gene product known as ALTO, or alternate Large T open reading frame, that arises from an alternative reading frame of LT (Figure 1A). Through phylogenetic analysis, ALTO appears to most closely resemble the murine polyomavirus middle T antigen, although these two proteins do not share sequence homology [65]. Thus far, ALTO does not appear to contribute to viral replication, and there is currently no evidence to suggest the presence of ALTO protein expression in MCPyV+ MCC tumors [65].

Late MCPyV viral gene products include the major capsid protein viral protein 1 (VP1) and the minor capsid protein 2 (VP2) (Figure 1A). VP1 forms pentamers that can self-assemble into icosahedral capsids and also mediates attachment to cell surfaces through interactions with sulphated polysaccharides and sialic acid [66,67]. VP2 mediates post-attachment viral entry and is necessary for native MCPyV infection. Unlike other polyomaviruses, MCPyV does not express a functional VP3 protein [68,69]. The MCPyV genome also expresses a 22-nucleotide viral miRNA (MCV-miR-M1) from the late strand that regulates LT expression levels [70].

#### 1.2.3. MCPyV Tropism

The cell type infected by MCPyV that produces progeny virus is still a matter of contention. Similar to other DNA tumor viruses like HPV [72], MCPyV was discovered in tumors in which the viral genome had integrated, and thus viral replication was thwarted. Therefore, the conditions involved in the discovery of MCPyV did not provide definitive insight into what cell types support the viral life cycle. Researchers were able to successfully sequence and assemble the full-length MCPyV genome from a patient-derived MCC tumor (MCC350) using sequential primers spanning the circular viral genome, paving the way for future studies [26].

The discovery of MCPyV in a cutaneous malignancy underscores the possibility that the skin and resident cell types therein may be natural sites of MCPyV infection. Indeed, the MCPyV virus is chronically shed from human skin in healthy adults and can be detected in skin swabs [53,56,73]. However, MCPyV genomic material has also been detected in non-cutaneous samples such as respiratory tracts [74], urine samples [75] and fecal matter [76]. Therefore, it is also possible that a non-cutaneous cell type is targeted for MCPyV infection.

Despite its discovery in MCC, mature Merkel cells within the skin are not thought to be the likely target cells of infection, even though normal Merkel cells and MCC share overlapping biomarkers. This is for several reasons. First, Merkel cells are exceedingly rare in human skin (reviewed in [77]). Given that MCPyV can be readily detected in skin swabs [73], it seems more likely that the virus infects a prominent cell type within the skin that supports replication at levels high enough to be readily detected in the skin. Second, Merkel cells are considered to be post-mitotic quiescent cells [78] and while not outside the realm of possibility, a viral infection of a terminally differentiated and non-proliferative cell seems unlikely to support robust replication.

Multiple studies have demonstrated MCPyV’s ability to infect various cell types in vitro and/or ex vivo. Initial studies by Neumann and colleagues used a synthesized recombinant MCPyV plasmid to evaluate the infectivity of several cell types [79]. Their studies showed three cell lines supported MCPyV replication: the primitive neuroectodermal tumor cell line PFSK-I, human non-small cell lung carcinoma cell line, H1299, and human embryonic kidney cells, HEK293. MCPyV infection of the PFSK-1 cell line, specifically, resulted in particles evident by electron microscopy; however, no secondary infections were detected, suggesting that these particles were not infectious. Schowalter et al. used MCPyV pseudoviruses to infect a wide variety of cell types in the NCI-60 panel of human cancer cell lines, as well as primary epithelial cells and the immortalized epidermal keratinocyte line HaCaT [80]. Cell lines from a variety of solid tumor types, melanoma cancer cell lines, and primary keratinocytes all supported infectious MCPyV entry.

Dermal fibroblasts have been the focus of multiple recent studies exploring MCPyV tropism and, to date, are the only cell type shown to support the full MCPyV life cycle in vitro, including infectious progeny production and secondary infections. Liu et al. used recombinant MCPyV to explore viral entry in a variety of human cell lines [81]. Primary human keratinocytes and fibroblasts derived from both adult and fetal tissue supported viral entry, yet only fibroblasts supported the full MCPyV life cycle that led to the generation of infectious progeny particles capable of secondary infections of uninfected cells. Likewise, Abere et al. also found that human foreskin fibroblasts (HFF-1 and BJ-hTert), along with bone marrow-derived human mesenchymal stem cells (MSC-bm) and adipose-derived MSCs (MSC-a), support MCPyV infection using a synthetic MCPyV circular viral genome (or minicircle) tagged with a fluorescent reporter [82]. Alternatively, another study found that MCPyV viral DNA replication causes genomic stress in fibroblasts, leading to cell cycle arrest and senescence [83], which is potentially counterintuitive to the hypothesis that this cell type is the source of progeny virus.

Collectively, these studies suggest that MCPyV can enter a variety of cell types in vitro, although conditions have only been achieved thus far that permit the full viral life cycle in fibroblasts. As will be discussed in subsequent sections, preclinical in vitro and in vivo models are helping to reveal further information about the tropism of MCPyV as well as its role in MCC development.

## 2. Role of MCPyV in MCC

### 2.1. MCC Cell of Origin

To establish the role of MCPyV in MCC and develop relevant models of this virus-induced cancer, it is important to consider not only MCPyV tropism but also the cell of origin of MCC, which may or may not be the cell type in which the virus replicates. There is limited evidence supporting Merkel cells as the cell of origin for MCC. Both normal Merkel cells and MCC cells share expression of the Merkel cell-specific biomarker, CK20, although staining in normal Merkel cells appears cytoplasmic [38], whereas MCC tumors have a more ‘dot-like’ appearance [38]. MCC cells express several other markers that normal Merkel cells do not, such as the adhesion molecules CD171 and CD24 and KIT receptor tyrosine kinase c-kit [84,85]. Merkel cells are located near the basal layer within the epidermis of human skin and in hair follicles, whereas MCC tumors arise primarily in the dermis [86]. Additionally, Merkel cells appear to be mitotically inactive [78], and this quiescent state seems to challenge the pre-requisite of precursor cancer cells [87]. Moreover, MCC does not occur in mouse models when Merkel cell-targeted transgene systems are employed [88,89].

Insight into a potential cell of origin for MCC may be gleaned through our knowledge of normal Merkel cell development. It is believed that a pluripotent population of cells differentiates to generate Merkel cells. Initially, Merkel cells were postulated to arise from the neural crest [90,91,92,93]. More recently, evidence from in vivo studies indicates that Merkel cells originate from epithelial progenitor cells following a cascade of intra- and intercellular signaling events and the action of multiple transcription factors [94,95,96,97,98]. In the skin of transgenic mice, Van Keymeulen et al. utilized a cell-lineage tracing method to label K14+ epithelial cells and track their development into Merkel cells [96]. In a subsequent study, the importance of a Merkel cell specification factor, atonal bHLH transcription factor 1 (ATOH1), was demonstrated to be of importance in Merkel cell development since its conditional deletion in K14+ cells in murine skin resulted in the absence of Merkel cell development [99]. In contrast, targeted deletion of ATOH1 in cells from the neural crest lineage did not affect Merkel cell numbers [99], further substantiating the theory that Merkel cells arise from epidermal progenitors and not the neural crest.

Considering this evidence, it can be argued that MCC cells also arise from the same population of cells from which Merkel cells arise. Supporting this hypothesis, recent research studying MCPyV+ MCC identified overlapping somatic mutations when performing genetic analysis of a tumor consisting of a benign trichoblastoma derived from the epithelial lineage and MCPyV+ MCC [100]. Additionally, an analysis of tissue-specific DNA methylation patterns revealed similarities between MCCs and epithelial cancers, including squamous cell carcinoma [101]. These shared molecular traits further support the hypothesis that MCCs, like Merkel cells, arise from epithelial progenitors in which the MCPyV viral genome had integrated prior to clonal expansion.

Other cell types have also been proposed as MCC cells of origin. Dermal fibroblasts have been proposed considering that a majority of MCC tumors develop in the dermal layer where fibroblasts reside [86]. As described earlier, fibroblasts are also the only cell type reported thus far to support the full MCPyV viral life cycle [81]. Furthermore, MCPyV ST has oncogenic properties in rat fibroblasts [102]. Other non-cutaneous cell types, such as B cells, have been proposed as a potential cell of origin based on the histological similarities between lymphoid malignancies and MCC [103] and the positive expression of pro-B cell markers TdT and PAX5 in MCC tumors [104,105]. This has led to the model that MCPyV infects early stages of B cells, thus initiating the development of a cancer with “B cell-like” traits that localizes within the dermal layer of skin [86]. Neuronal precursor cells are also potential candidates based on a recent report that described the acquisition of neurofilament-like characteristics in MCPyV+ MCC cells upon T antigen knockdown [106]. Given the lack of clarity regarding MCPyV tropism and MCC cell of origin, the development of in vitro and in vivo models to explore MCPyV entry and T antigen expression in various cell types is a vital step towards our understanding of MCPyV+ MCC.

### 2.2. Viral Genome Integration

Integration of the MCPyV genome into the host genome is likely a critical event in tumor initiation and the oncogenic process of MCC development [26,107,108]. Importantly, these integration events are clonal within any given MCPyV+ MCC, suggesting they occurred at an early stage in the tumorigenesis process, consistent with a causal role of the virus in the development of MCC. In MCC tumors, MCPyV viral genome integration results in the loss of the late region and retention of early region sequences with an average viral copy number of 5.2 (range 0.8–14.3) copies per cell [50]. The integration patterns of the MCPyV genome result in constitutive expression of two viral proteins, small T antigen (ST) and a truncated form of large T antigen in MCPyV+ MCCs [50,109,110].

### 2.3. MCPyV T Antigen Expression and Oncogenic Properties

Expression of the viral LT and ST proteins is required for the continued growth of MCC-derived cell lines, providing evidence that these viral genes contribute to MCC [111]. This addiction of MCC cells to MCPyV T antigens is a hallmark of viral-induced oncogenesis. Next-generation sequencing (NGS) clearly distinguishes MCPyV− MCC and MCPyV+ MCC as two distinct tumor subtypes and provides further evidence that the MCPyV T antigens function as oncogenic drivers in MCPyV+ MCC [107,112]. MCPyV− MCC has a significantly higher mutational burden compared to its counterpart. Many of these mutations resemble UV-induced DNA mutations, which can decrease the DNA damage response and activate signaling pathways involved in the development of other cancer types of the skin. MCPyV− MCCs also consistently harbor mutations that inactivate the tumor suppressor genes *RB1* and *TP53* [113,114,115], suggesting that these mutations and their downstream effects are the primary drivers of oncogenesis in MCPyV− MCC. In contrast, MCPyV+ MCCs have greater genomic integrity with very few somatic mutations relative to MCPyV− MCCs, including intact coding regions for the tumor suppressor genes *RB1* and *TP53.* Data indicate that MCPyV LT and ST antigens target the pRb and p53 tumor suppressor proteins, respectively, for inactivation, as described below. These observations provide evidence to support the hypothesis that MCPyV and viral T antigen functions, rather than mutational burden, are the main oncogenic drivers in MCPyV+ MCC, though it should be recognized that MCCs also preferentially arise at sun-exposed sites, regardless of viral status [40]. There are several proposed mechanisms for how the MCPyV T antigens facilitate MCC development, discussed in this section.

#### 2.3.1. MCPyV LT

The MCPyV LT antigen is one of the two viral proteins found to be frequently expressed in MCPyV+ MCC cells. Within integrated sequences of the MCPyV early region in MCC tumors, mutations are found in the LT coding sequence that lead to the expression of truncated forms of LT that consistently lack the C-terminal helicase domain yet retain the LXCXE domain [26,109]. These truncated LT proteins lack the functional domains needed to catalyze viral replication yet retain the ability to inactivate the tumor suppressor pRb (Figure 1B). The loss of C-terminal regions in LT also confers oncogenic properties. In vitro studies show that expression of full-length LT induces a DNA-damage response and cell cycle arrest in a p53-dependent manner that is detrimental to cellular proliferation [116]. On the other hand, truncated LT attenuates the repair of UV-induced DNA damage [64]. These findings and their downstream effects were clearly shown in the report by Cheng et al. [23], whereby the expression of truncated LT was shown to promote cell growth, while expression of the C-terminal domain alone, or full-length LT, negatively regulated cell growth.

The retention of the LXCXE motif within truncated LT (Figure 1B) allows association between LT and the cellular tumor suppressor pRb [24,109]. This interaction results in enhanced E2F-transcriptional activity and promotes growth in MCPyV+ MCC cells [24,117] and also enhances entry into S-phase, cellular proliferation, and motility in in vitro studies [118]. We also recently demonstrated the importance of the truncated LT/Rb interaction in vivo in cutaneous phenotype development and tumorigenesis in MCPyV transgenic mice [119]. Unlike LT proteins of other polyomaviruses, such as SV40 LT, MCPyV LT does not appear to possess the ability to inactivate p53. On the contrary, the LT/pRb association has been shown to lead to upregulation of ARF, which inhibits the activity of the E3 ubiquitin-ligase MDM2 that targets TP53 for degradation and subsequently increases p53 levels [25,120]. The inactivation of pRb by the MCPyV LT protein is functionally equivalent to the inactivation of pRb by mutations present in MCPyV- MCC [113].

#### 2.3.2. MCPyV ST

The ST antigen is also expressed in MCPyV+ MCCs. MCPyV ST is currently considered the primary transforming oncoprotein of MCPyV, and it possesses transformation capabilities independent of LT expression, as seen in rat fibroblasts and epithelial cells in vitro and in vivo [89,121,122,123,124]. There are multiple proposed mechanisms for the oncogenic potential of MCPyV ST. Several groups have demonstrated that ST induces phosphorylation of the cellular translation factor eIF4E binding protein 1, 4E-BP1, through mTORC1 to support active cap-dependent translation and gene expression [102,125,126]. ST can also alter cellular gene expression at the transcriptional level by recruiting an MYCL-MAX heterodimer to the EP400 complex histone acetyltransferase complex [127]. Cheng et al. showed that this ST/MYCL/MAX and EP400 interaction was necessary to maintain cell viability in MCPyV+ MCC cell lines in vitro. Notably, genes encoding lysine-specific histone demethylase 1A (LSD1) and mouse double minute 2 homolog (MDM2) were upregulated, which were necessary for MCC cell survival in vitro and MCC xenograft growth in vivo [25,36,128]. Gupta et al. have also reported that ST attenuates the expression of the tumor suppressor N-myc downstream-regulated gene 1 (NDRG1), causing dysregulation of the cell cycle [129].

ST has also been reported to activate several oncogenic signaling pathways. ST expression promotes cellular growth in vitro by inducing phosphorylation of c-Jun downstream of the MEK/ERK signaling cascade [130], increasing glycolytic activity [131] and disrupting inter-cellular junctions [132]. ST expression has also been associated with activation of the non-canonical NF-κB pathway, induction of a senescence-associated secretory phenotype (SASP), and enhanced MCC cell proliferation [133].

Interestingly, MCPyV ST appears novel compared to other polyomaviruses with respect to the role the interaction of MCPyV ST antigen with the PP2A Aα subunit plays in cellular transformation [134]. Although MCPyV ST antigen possesses the ability to bind to the PP2A Aα subunit in co-immunoprecipitation assays [102], this interaction appears to be dispensable for MCPyV ST-mediated transformation of cells in vivo [122]. However, this interaction was still involved in the cellular proliferation of MCPyV+ MCC cells.

While both truncated LT and ST have unique functions implicated in oncogenesis, their combined expression and functional interplay are likely important for MCPyV-mediated oncogenesis. As one example, this dynamic is apparent in the context of p53 inactivation. Not only does singular LT expression fail to attenuate p53 activity, but rather it appears to upregulate p53 activity [25]. However, this LT-mediated upregulation of p53 activity was dampened when ST was co-expressed in the same cells. In this study, depletion of EP400 in MCPyV+ MCC cell lines led to increased p53 target gene expression. ChIP and RNA-sequencing analysis performed on EP400-depleted cells identified target genes of the ST/EP400 complex, including MDM2 as well as evidence for the activation of MDM4, another p53 regulator. The upregulation of MDM2 and MDM4 are well-known mechanisms that suppress p53 activity (reviewed in [135]). Consequently, cells that harbor the MCPyV T antigens can bypass p53-mediated DNA-damage responses by upregulating MDM2 and MDM4 expression, thus revealing a mechanism by which the MCPyV T antigens inactivate the cellular tumor suppressor p53 through indirect means. Therefore, similar to other well-known human DNA tumor viruses, the MCPyV truncated LT and ST antigens target both cellular tumor suppressors pRb and p53 for inactivation in MCCs to promote cell survival and transformation. The complementary functions of truncated LT and ST may also extend to other oncogenic functions that contribute to the development of MCC.

### 2.4. MCPyV-Associated Cellular Plasticity and/or Reprogramming

There is growing evidence that the MCPyV T antigens possess some capacity to promote cellular plasticity and/or reprogramming in different cell types, leading to the hypothesis that T antigens promote transdifferentiation of infected cells into cells with Merkel cell phenotypes that then give rise to MCC. One line of evidence to support this hypothesis is the observation that the MCPyV T antigens can induce neuroendocrine gene expression and characteristics within potential MCC precursor cells. For instance, MCPyV LT upregulates the expression of SOX2, a specification factor involved in the differentiation and maturation process of normal Merkel cells [94,95,106]. LT also stabilizes ATOH1 protein expression in epithelial cells [136]. ST can also induce expression of the neuroendocrine markers (and MCC markers) RCOR2 and INSM1 in vitro by recruiting EP400 complex to the promoter region of these genes [36,137]. The ability of the MCPyV T antigens to increase the expression of this collection of genes may allow certain permissive cell types to acquire Merkel cell or MCC-like traits. As will be discussed in subsequent sections, in vitro and in vivo models of MCPyV+ MCC will prove useful in determining how and to what extent the MCPyV T antigens contribute to cellular plasticity in different cell types.

## 3. Considerations for Designing MCPyV+ MCC Models

There are several considerations when generating models of MCPyV+ MCC. Generally, models should incorporate conditions that closely replicate the cellular environment and include factors that have been established as playing a role in MCPyV+ MCC development. The source of MCPyV genomic DNA and/or proteins is important to consider, as one would likely want to use MCC-derived viral DNA (i.e., one containing truncated LT) for generating models of MCPyV+ MCC but an MCPyV isolate derived from normal skin (i.e., a WT isolate with intact LT) for MCPyV replication studies. Given that various LT truncation points have been identified in patient-isolated MCC samples [23,109], one might consider particular truncation patterns should it be determined that different truncated LT proteins confer different effects on cells/tissues. The spatial and temporal expression of viral proteins and cellular cofactors should also be considered. For instance, the cell types used or tissues targeted in vitro and in vivo, respectively, should reflect advances in our knowledge regarding MCPyV tropism and MCC cell of origin. The temporal expression of the MCPyV T antigens can also be modulated to mimic host conditions wherein MCC arises. Overall, as further information is learned about MCPyV+ MCC and potential biological and/or environmental cofactors involved, in vitro and in vivo models can and should be adapted in an attempt to recapitulate these conditions.

## 4. In Vitro Models of MCPyV Infection & MCPyV+ MCC

**Figure 2:**
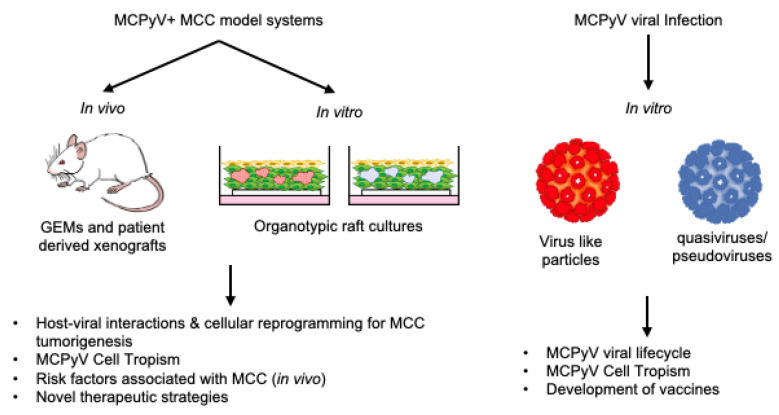
In vitro and in vivo biological model systems for MCPyV infection and MCPyV+ MCC research and their utilities.

In vitro model systems utilizing human cells are advantageous tools that allow studies of viral entry and replication, as well as the study of MCC cell lines, without the need for a suitable live host (Figure 2). This section discusses the current state of in vitro models being used to study several facets of MCPyV and MCPyV+ MCC, including MCPyV tropism and infection, MCC cell of origin, and MCPyV+ MCC biology within three-dimensional human skin equivalents.

### 4.1. Models of MCPyV Tropism and Infection

A particular challenge in developing models of MCPyV replication is the lack of naturally isolated and infectious MCPyV particles. Several groups have developed molecular tools combined with in vitro tissue culture systems to address this limitation. One of the more common approaches used to overcome the lack of natural MCPyV virus stocks has been to generate MCPyV virus-like particles (VLPs), pseudoviruses, and quasiviruses. These in vitro-generated alternatives have enabled researchers to perform in vitro studies to explore MCPyV tropism and the temporal dynamics of the virus life cycle. Virus-like particles (VLPs) are composed of late structural proteins (VP1, VP2) that naturally self-assemble into viral particles [138,139,140]. VLPs have proven to be powerful tools that can be used as gene transfer vectors or vaccine components, as was the case with HPV (reviewed in [141]). Pseudoviruses are VLPs that contain genetic material, most frequently a gene that encodes a reporter molecule such as a fluorescent protein (GFP, RFP, etc.) [142,143] and that have been used to study cellular tropism, species-specific restrictions, and viral entry mechanisms of other DNA tumor viruses [144,145,146,147,148]. Quasiviruses are virus-like particles that contain the complete viral genome and are generated in vitro instead of being produced under natural conditions in vivo [69,149,150]. Generally, large amounts of the viral genome are liberated from a bacterial expression vector, linearized, and re-ligated to generate circular plasmids. These circular plasmids are transfected into cells expressing late viral proteins involved in virus assembly to produce native virions in a process that is often less efficient and robust than natural virus production. Quasiviruses are a close substitute for natural infectious virus particles and can complete the full virus life cycle under the correct conditions. As will be discussed in this section, several groups have used MCPyV VLPs, pseudoviruses, and quasiviruses to generate in vitro infection models.

Schowalter et al.’s studies used pseudoviruses synthesized from MCPyV VP1 and VP2 genes conjugated to a fluorescent reporter to identify the cellular receptor utilized for MCPyV viral entry [149]. Their studies identified a viral entry mechanism utilizing sulfated glycosaminoglycans for attachment and entry, which is reminiscent of the viral entry of papillomaviruses and hints at MCPyV’s potential natural tropism in human skin [148,151].

Liu et al. used MCPyV pseudoviruses and quasiviruses to demonstrate dermal fibroblasts’ capacity for MCPyV viral entry and viral replication [81]. In this study, epithelial and dermal cells were isolated from human foreskin and infected with MCPyV pseudoviruses carrying a GFP reporter, similar to the approach used to infect the NCI-60 panel of cells in the Schowalter et al. [80] study. Both primary epithelial cells and dermal fibroblasts supported viral entry. When MCPyV quasiviruses were used to infect cells, only dermal fibroblasts showed LT or VP1 expression, indicative of MCPyV viral gene expression. Supplementation of dermal cell cultures with epidermal (EGF) and fibroblast growth factors (FGF) promoted viral entry by upregulating the expression of matrix metalloproteinase (MMP) proteins. In the same study, ex vivo infection assays of human-derived foreskin and scalp samples were performed, and the virus was detected in fibroblasts within the dermal layer proximal to hair follicles [81]. The model proposed by Liu et al. suggests that MCPyV viral entry is facilitated to some extent by a more accessible dermal structure. Since in vitro studies can often highlight areas to explore using in vivo models, it would be interesting to target MCPyV T antigen expression to murine skin with a fibroblast-specific promoter in light of these results.

Neumann et al. also developed an in vitro model of MCPyV replication using a consensus viral genome that was generated by re-assembling an MCPyV genome based on the sequences derived from MCC tumors publicly deposited in NCBI [79]. Although this viral genome was re-constructed based on the incomplete genome seen in diseased tissue, a comparison of this synthetic viral genome with the wild-type full-length MCPyV sequence published later by Schowalter et al. showed that the sequences were identical and, therefore, would express the same viral proteins that wild-type MCPyV would. The synthetic genome was transfected into several cell lines, and viral gene expression was monitored post-transfection. As mentioned above, viral replication was seen in numerous cell types, although no infectious viral particles were generated.

More recently, Abere et al. designed and generated another version of a circular viral genome for MCPyV [82]. Using a recombinase-mediated minicircle system, an MCV minicircle (MCVmc) was generated, which has higher yield efficiency than traditional recircularization approaches generally used. Moreover, the group successfully introduced previously known mutations into the MCVmc construct that attenuate specific host interactions, allowing for the study of their respective effects on viral replication. They also included a fluorescent reporter that allows real-time observation of the kinetics of virus replication. Their study also reported viral entry and replication in various cell types; however, transfection of the MCVmc construct tagged with a fluorescent reporter also failed to produce infectious particles for secondary infections, and complementation with other expression plasmids encoding VP1 and VP2 was required for single-round transmission assays in vitro.

Collectively, these in vitro models are providing significant insight into potential cellular tropism and the dynamics of viral replication and will undoubtedly continue to shed light on this important aspect of MCPyV virology.

### 4.2. In Vitro Models to Explore MCC Cell of Origin

As previously discussed, the cellular origin of MCC has yet to be defined. Models using human cell lines or MCPyV+ MCC cell lines have been used in efforts to identify this cell type. For example, Kervarrec et al. utilized lentiviruses encoding MCC tumor-derived MCPyV T antigens (ST and truncated LT) to transduce primary human keratinocytes. This was also done in conjunction with a host factor involved in Merkel cell development, GLI family zinc finger 1 (Gli1) [136]. Singular expression of the MCPyV T antigens induced morphological changes in keratinocytes as well as expression of the early Merkel cell markers K8 and K18, indicating that the T antigens have some degree of re-programming capacity. In keratinocytes expressing the MCPyV T antigens and GLI1, advanced Merkel cell phenotypes such as a floating cell morphology (consistent with non-adherent MCPyV+ MCC cell lines) and expression of Merkel cell markers SOX2, K8, and K20 were observed.

Another independent study by Harold et al. developed a T antigen knockdown model in an attempt to elucidate the precursor cell for MCPyV+ MCC [106]. MCPyV T antigens were knocked down in the MCPyV+ MCC cell lines MS-1, CVG-1, and MKL-1, followed by co-culture with keratinocytes to recapitulate the cellular environment where MCCs occur. Upon T antigen knockdown, these cells developed neurite cell-like projections, and Merkel cell-related gene expression (*ATOH1*, *SOX2*, *HES6* and *KRT20*) was decreased. Interestingly, the adoption of the neuronal-like phenotype was dependent on co-culture with keratinocytes, suggesting that intercellular crosstalk is required to facilitate this trans-differentiation process. Exogenous expression of truncated MCPyV LT antigen in MCPyV+ MCC cells with LT knockdown restored SOX2 and, in turn, ATOH1 expression and decreased neurite-like outgrowth, all effects that were dependent on the pRb-binding ability of LT. These results suggest a mechanism whereby MCPyV enters and integrates into a neuronal precursor cell, and viral T antigen expression promotes trans-differentiation into an MCC-like cell.

As we gain more knowledge about MCPyV and MCPyV+ MCC, other cell types may be proposed as the MCC cell of origin. Therefore, future in vitro models using other human cell types, such as fibroblasts or B cells, as mentioned earlier, would help elucidate the identity of the MCPyV+ MCC cell of origin.

### 4.3. Organotypic Raft Models of MCPyV+ MCC

An ideal in vitro model to study MCC, a cutaneous malignancy, would incorporate common resident skin cell types and also recapitulate the three-dimensional (3D) spatial dynamics of human skin. These important traits can be achieved through the use of organotypic raft cultures. Organotypic “raft” cultures, also known as skin equivalents, recapitulate the stratified squamous epithelium and the dermal component of the skin in a 3D in vitro setting [152]. Keratinocytes are grown on a dermal equivalent containing fibroblasts embedded in collagen, and these cultures are then raised to the air-liquid interface, which drives stratification and terminal differentiation in a process similar to what occurs in the skin. The resulting rafts histologically resemble human skin with a cornified epidermal equivalent on top of a dermal equivalent. Our lab has used this in vitro model system to study the epithelial-tropic human papillomaviruses (HPVs), the life cycle of which is tied to the terminal differentiation process found within the epidermis [153,154,155]. However, rafts are limited in terms of fully replicating the human skin environment, as they lack diversity of cell types, vascularization, and immune cell infiltration. Nevertheless, rafts are an in vitro platform to study cellular interactions and crosstalk between cell types and potential therapeutic agents in the context of a 3D human skin equivalent.

**Figure 3:**
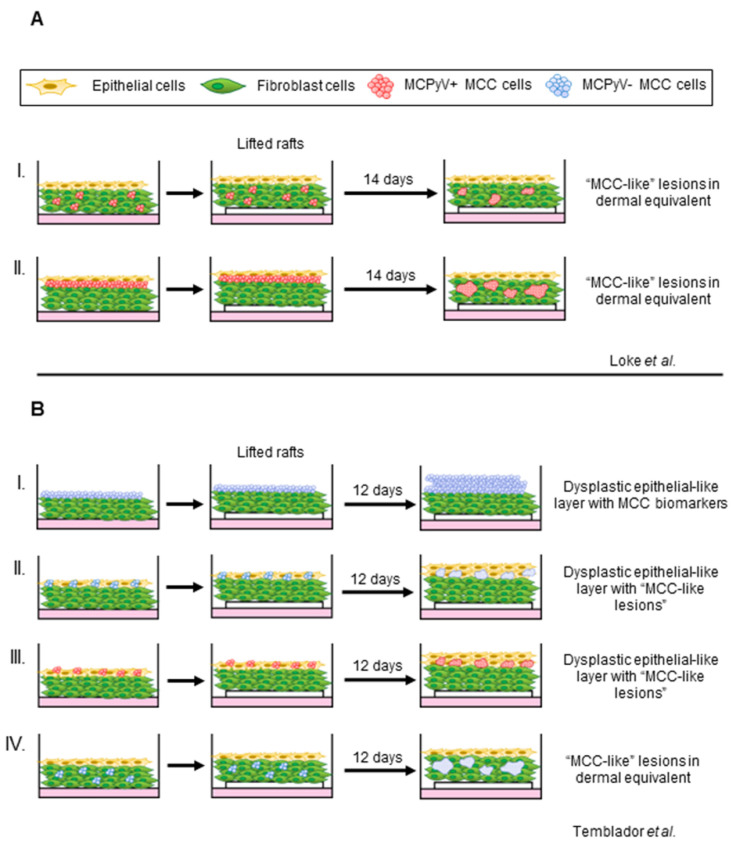
Graphical summary of current in vitro 3D tissue culture system generating MCC phenotypes. (**A**) I. MCPyV+ MCC cells (red) embedded within the dermal equivalents consisting of fibroblasts (green) or II. as a single transition layer between the dermis and the epithelial layer (yellow) produced MCC-like lesions within the dermal layer (Loke et al. [156]). (**B**) MCPyV- MCC cells (blue) grown adherent on the dermal equivalent result in a dysplastic epithelial layer with positive detection of MCC biomarker. II, III) When co-cultured with epithelial cells, nodules containing “MCC-like” cells were observed in the epithelial layer. IV) Only MCPyV- MCC (not MCPyV+ MCC) cells grown with fibroblasts in the dermal equivalent produce phenotypes consistent with those seen in human MCC ([Temblador et al. [157]).

We and others have developed models using organotypic rafts to study MCPyV+ MCC [156,157] (Figure 3). We adapted our laboratory’s established protocol for studying the HPV life cycle by incorporating the MKL-1 cell line to develop a raft-based model for the co-culture of MCPyV+ MCC cell lines [155,158]. Our studies identified an optimal co-culturing method in which MCPyV+ MCC cells are suspended in a layer of collagen (Figure 3A). Within the organotypic rafts, MCC-derived lesions developed within the dermal layer that recapitulated the histopathology of human MCC. Features of MCPyV+ MCC cells were preserved, as these cells remained proliferative and displayed biomarkers of MCC [24]. The development of MCC-like lesions from MCPyV+ MCC cells in organotypic rafts was not dependent on the presence of fibroblasts or keratinocytes. However, further efforts to explore or characterize potential changes in the MCC lesions present in rafts under these conditions are ongoing.

Temblador et al. also recently reported an organotypic raft-based model for MCC that included both MCPyV- (MCC13, MCC12/2 and MCC26) and MCPyV+ MCC (WAGA, MS-1, and MKL-1) cell lines [157]. In this model, MCC cells were either added directly on top of the dermal equivalents, mixed with keratinocytes at different ratios before seeding, or co-cultured with fibroblasts in the dermal equivalent (Figure 3B). Interestingly, intraepidermal MCC-like lesions developed with MCPyV+ MCC cell lines within these rafts and not in the dermal equivalents when co-cultured with fibroblasts, which differs from our results [157]. These discordant results may reflect different rafting techniques, the use of different keratinocyte/fibroblast cells, and/or the heterogeneity of patient-derived MCC cell lines used in the two studies [108,158,159,160]. Using this in vitro system, Temblador et al. observed significantly higher expression of several genes associated with tumorigenesis in cells grown within the rafts, such as extracellular matrix, cell adhesion molecules, and growth factors, compared to cells cultured in a monolayer [157]. This finding highlights the potential biological importance of using organotypic rafts, whereby cell-cell interactions can be elucidated in a biologically relevant context.

Because these raft models mimic human skin biology and structure, they are powerful tools potentially capable of recapitulating the entire spectrum of virus-mediated disease in vitro, from infection to potential cellular transformation. Future studies could combine MCPyV quasiviruses to test the ability of stratified squamous epithelium to support the viral life cycle. Fluorescent reporters such as Abere et al.’s MCVmc could be used to track progeny virion production throughout the raft. Furthermore, host cells and MCPyV+ MCC can be genetically manipulated to test the role of host factors in viral pathogenesis and to reveal potential functions that may not be apparent in a monolayer culture system. Organotypic raft MCPyV+ MCC co-culture models can also serve as important preclinical platforms to efficiently test therapeutic targets and pharmaceutical agents as they emerge.

## 5. In Vivo Models of MCPyV+ MCC

In this section, we discuss the current state of in vivo models used to study MCPyV+ MCC. The gaps in our understanding of natural MCPyV infection and the restrictive tissue and species tropism of polyomaviruses have resulted in a current lack of in vivo models that recapitulate natural MCPyV infection. Nonetheless, the model systems discussed in this section have been used to further our understanding of MCPyV interactions with the skin and MCPyV+ MCC development (Figure 2). In vivo model systems share the same advantage as in vitro models in terms of genetic tractability. However, because these model systems utilize a live host, key biological processes, such as angiogenesis and interactions from the cellular microenvironment, can be studied. In addition, in vivo model systems can also be expanded to include co-factors linked to tumor initiation or promotion, such as chemical carcinogens or environmental factors such as UV.

### 5.1. Chorioallantoic Membrane (CAM) Models of MCPyV+ MCC

One in vivo model for MCC involves the use of chick Chorioallantoic Membrane (CAM). CAM was first described by Murphy and Rous as a model system for tumor growth and development, wherein they demonstrated the growth of Rous sarcoma transplanted onto the CAM [161]. CAM has now been widely used to study a variety of human cancer cell types as well as cancer-related biological processes such as hypoxia and testing of anti-cancer drugs [162,163] (reviewed in [164]). In fertilized avian eggs, during embryogenesis, the mesoderm layer of the allantois fuses with the mesodermal layer of the chorion to form the chorioallantoic membrane. The CAM possesses an extensive vascular network, which not only facilitates gas exchange for the developing embryo but also supplies necessary growth nutrients (reviewed in [165]). The embryo itself is immunosuppressed, creating a favorable biological environment to support the growth of grafted tumor cell lines from other species. CAM assays are an attractive alternative to xenografts or GEMs as they take significantly less time for grafted tumor cells to achieve vascularization within the CAM [166].

**Figure 4:**
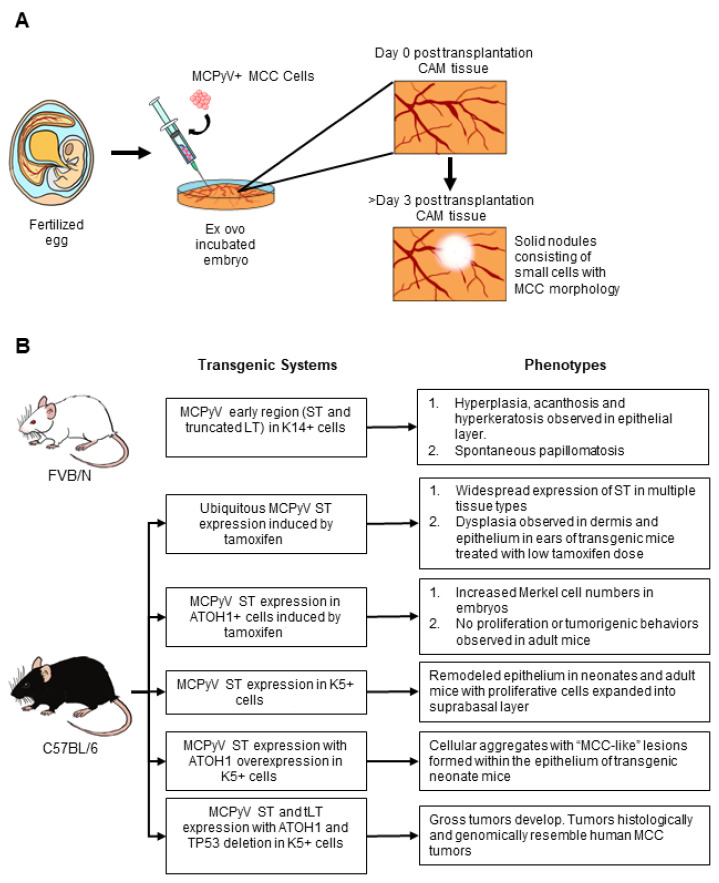
In vivo models for MCPyV+ MCC. (**A**) Short-term MCPyV+ MCC in vivo model with chick chorioallantoic membrane (CAM) assay as described in Bhat et al. [167]. Fertilized eggs were transferred for ex ovo incubation in dishes after 3 days. The embryos are then incubated for an additional 7 days. MCPyV MCC cells were then transplanted onto the vascular structure on the CAM and incubated for 3–7 days. Solid nodules can be observed on the CAM tissues 3 days post-transplantation. (**B**) Graphical summary of current genetically engineered mice (GEM) for MCPyV+ MCC (Shuda et al. [89]; Verhaegen et al [123,124,137]; Spurgeon et al. [173]).

To model MCPyV+ MCC using CAM, Bhat et al. grafted 3 different MCPyV+ MCC cell lines (MKL-1, WaGA and PeTA) onto fertilized eggs (Figure 4A) that had been incubated ex ovo [167]. Tumor nodules with extensive vascularization were visible on Day 3 post-grafting, and MCC cells invaded surrounding CAM tissue causing epithelial remodeling, suggesting that this in vivo model may recapitulate the 10% of intraepidermal MCC cases observed in humans. Clusters consisting of small, rounded cells, a morphology consistent with that of MCCs, expressed MCC biomarkers cytokeratin 20 (K20) and chromogranin A (CGA), as well as MCPyV LT. CAM models, therefore, are an attractive method to grow MCPyV+ MCC samples in an organoid-like structure that conserves cell-cell interactions. In addition, this CAM model is a relatively inexpensive in vivo model that can be used to test therapeutic compounds within a relatively short time frame compared to animal studies.

### 5.2. Xenografts and Patient-Derived Xenografts

Patient-derived xenografts (PDXs) have been developed for MCPyV+ MCC cells. In PDX models, tumor cells are introduced either subcutaneously or intravenously into immunodeficient mice [168,169]. The lesions that developed in these mice exhibited histological traits resembling those of human MCC, retained expression of ST and truncated LT, and also resulted in lung metastases. This metastatic pattern is consistent with those reported in metastatic MCC cases in humans [27].

MCC xenografts have been used to validate new therapeutic strategies [170], an invaluable contribution for such an aggressive disease [47,48,171]. Demonstrating the utility of MCC xenograft models, Fang et al. tested copanlisib, a PI3K inhibitor, as a potential treatment for advanced MCC [170]. Aberrant PI3K signaling is often observed in MCC, regardless of MCPyV status [172]. After confirming increased PI3K signaling in two MCPyV- and one MCPyV+ MCC patient-derived cell line, they found copanlisib inhibited cell proliferation and induced apoptosis. This drug was also effective in PDX tumors with no adverse effects on the health of the mice. Xenograft studies have also revealed the efficacy of targeting important genes during MCC tumorigenesis, such as LSD1 [36,128], and even to re-activate TP53 response to induce cellular death in MCC cells [25].

While undoubtedly useful as a tool to study advanced stages of MCPyV+ MCC and to test therapeutic compounds, a caveat to PDX mouse models is that immunodeficient mice are used to allow transplantation and growth of MCPyV+ MCC cells without risking host rejection. Therefore, any studies exploring immunological response and immune cell infiltration are compromised in these mouse models.

### 5.3. Genetically Engineered Mouse Models of MCPyV+ MCC

Genetically engineered mice (GEM) are a valuable platform for studying various aspects of virus-associated disease, including interactions between viral and host factors, intercellular signaling, and the effect of the cellular microenvironment on the disease development process. Multiple GEM models have been generated as in vivo models for MCPyV+ MCC (Figure 4B). Specifically, GEMs using both conditional (using Cre recombination) and inducible transgenic systems (using tamoxifen or tet-inducible systems) have been created to mimic conditions necessary for the initiation and progression of virus-induced MCC. As discussed in this section, current GEMs for MCPyV+ MCC involve the expression of the MCPyV T antigens in cell populations considered to be cells targeted for MCPyV infection and/or MCC cells of origin. Since the laboratory mouse is genetically tractable, both gain-of-function and loss-of-function genetic mutations can be introduced into host genes to evaluate the potential role of host factors that may contribute to MCPyV T antigen-mediated transformation.

#### 5.3.1. MCPyV T Antigen GEMs

Several groups have generated in vivo MCPyV transgenic mouse models. Shuda et al. generated a GEM expressing only MCPyV ST [89]. Their GEM was generated on the *C57BL/6J* genetic background and expressed a transgene with a codon-optimized MCPyV ST sequence cloned downstream of the ubiquitous ROSA26 promoter. To achieve inducible MCPyV ST expression, Cre recombination was induced through treatment with tamoxifen. Ubiquitous ST expression at high levels at various anatomical sites, as driven by the ubiquitin C promoter Cre (Ubc-Cre), caused a rapid deterioration in the health of mice within 5 days post-tamoxifen treatment. Dysplasia resulting from hyperproliferative cells was observed in the dermis and epithelium of ears. When ST was expressed in Merkel cells using an ATOH1-driven Cre, there was an increase in the Merkel cell progenitor population, but no gross phenotypes or MCC-like pathology was noted. These findings could be interpreted as an argument against Merkel cells as the cell of origin for MCC.

Our lab and that of Dr. James DeCaprio utilized GEMs to demonstrate the tumorigenic properties of MCC tumor-derived MCPyV T antigens [173]. Our GEM was generated on the *FVB/N* genetic background, which is more susceptible to cutaneous malignancies due to a genetic polymorphism in the *Ptch1* gene [174,175]. The MCPyV early region derived from a MCC tumor specimen encoding both ST and truncated LT was cloned downstream of the ubiquitous *Rosa26* promoter with a lox-stop-lox cassette to allow for Cre-induced expression of the viral genes. Expression of the MCPyV early region was induced by Cre recombinase driven by the human K14 promoter, directing T antigen expression to the proliferative cells localized in the basal layer of the epithelium. In these MCPyV transgenic mice, we observed several gross phenotypes, such as hyperplastic skin, as early as postnatal days 8–10. Nearly half of the MCPyV T antigen-expressing mice spontaneously developed benign cutaneous tumors that varied in appearance (both smooth and exophytic). Our histological and immunodetection analyses revealed epithelial hyperplasia as well as reorganized protein expression of the E2F-responsive gene MCM7 throughout the full thickness of the stratified epithelium, which has frequently been used as a biological indicator of pRb inactivation [176]. However, despite these significant MCPyV T antigen-induced phenotypes, no lesions resembling MCC were observed, nor were any MCC biomarkers detected in the transgenic epithelium of these MCPyV transgenic mice.

We have since utilized our GEM model to further interrogate MCPyV T antigen functions in the murine skin. Using a multi-stage model of skin carcinogenesis, we explored the role of MCPyV T antigens in skin tumorigenesis [177]. Through the topical application of the chemical carcinogens that function as a tumor initiator (DMBA) or a tumor promoter (TPA), we defined the role of T antigens in these discrete stages of carcinogenesis. MCPyV T antigens synergized exclusively with the tumor initiator DMBA to cause tumor development, suggesting that the viral proteins function as tumor promoters in murine skin. We also observed a higher frequency of skin tumors and more advanced disease when both DMBA and TPA were applied to MCPyV transgenic mice, indicating that MCPyV can synergize with chemical carcinogens. Interestingly, the MCPyV T antigens behaved very similarly to the well-validated HPV16 oncogenes expressed in the skin in this same assay, underscoring the parallel functions of DNA tumor virus oncoproteins.

We have also explored the role of MCPyV LT binding to pRb in the development of cutaneous phenotypes in murine skin. In our GEM model, we generated mice that expressed both ST and truncated LT on a wild-type pRb background or a mutant pRb background [119]. This mutant Rb (Rb^ΔL^) lacks 3 residues necessary for interactions with LXCXE-containing proteins, including MCPyV LT and other DNA tumor virus proteins such as HPV E7, but does not interfere with other functions of pRb [178,179,180,181,182]. In this study, we found that the majority of phenotypes observed in MCPyV T antigen transgenic mice were significantly reduced in mice expressing the Rb^ΔL^, suggesting that the MCPyV truncated LT-Rb interaction plays a critical role in conferring phenotypes to epithelial cells expressing the MCPyV T antigens. However, although greatly diminished, we still observed some epithelial hyperplasia and increased protein expression of the E2F-inducible gene, MCM7, in the skin of MCPyV/ Rb^ΔL^ mice. These findings suggest that other LT functions, and/or those of ST also expressed in this transgenic mouse model, likely contribute to cutaneous phenotypes induced by the MCPyV T antigens in the skin of mice.

Extensive and seminal work has been done by Verhaegen et al. to develop GEMs for MCPyV+ MCC [123,124,137]. In their earliest study, MCPyV ST was constitutively expressed using a bovine Keratin 5 (K5) promoter to drive the expression of ST in K5+ epithelial cells [123]. In preterm transgenic neonates, significant epithelial remodeling and proliferation were observed in K5+ cells. Not only did these cells express increased levels of the proliferation markers Ki67 and pHH3, but the apoptosis marker also cleaved caspase 3 (CC3). This finding was also consistent in adult transgenic mice, which used a Cre-inducible system to drive the expression of ST in K5+ cells upon treatment with tamoxifen. However, no lesions resembling human MCC were observed in either neonatal or adult epithelia. They have since expanded their in vivo model system to incorporate several host factors, as described in the next section.

#### 5.3.2. Combined Host Factor and MCPyV T Antigen GEMs

In the GEMs that only express MCPyV T antigens, there has thus far been no development of disease that resembled MCPyV+ MCC, although several phenotypes consistent with neoplastic progression were observed. This prompted studies exploring the expression of other host factors in conjunction with MCPyV T antigens that may contribute to the development of MCPyV+ MCC.

Verhaegen et al. showed that the combined overexpression of transcription factor ATOH1 with MCPyV ST in K5+ cells produced cellular aggregates that histologically resembled human MCC lesions in the epithelium of transgenic neonate mice [124]. This phenotype was only observed in the combination of both ATOH and ST; in the same study, the expression of truncated LT did not exacerbate this phenotype, nor did ATOH1 overexpression alone cause lesions to form. Immunohistochemistry analysis showed that these lesions were positive for MCC biomarkers K20 and K8, and the cells were mitotically active, unlike normal mature Merkel cells. Unfortunately, the neonates arising in this model were not viable, limiting its use. Together with Shuda et al.’s report [89], these studies clearly indicate that MCPyV ST has oncogenic potential.

A subsequent model demonstrated, for the first time, de novo development of MCC in adult MCPyV transgenic mice [137]. Using a combination of Cre recombinase and Tet-inducible systems, expression of both the MCPyV T antigens and ATOH1 were driven in K5+ epithelial cells. In mice that expressed MCPyV T antigens with ATOH1 overexpression (small T antigen, truncated large T antigen, ATOH1 overexpressing (*SLA*) mice), no overt tumors were observed; however, similar to their previous report, small cell aggregates that were positive for K8 arose within the hair follicles of these mice. Hemizygous deletion of TP53 was induced in the same cells expressing the MCPyV T antigens and ATOH1 using a coding sequence of TP53 flanked by *loxP* sites. In these mice (small T antigen, truncated large T antigen, ATOH1 overexpressing, TP53/TP53Δ (*SLAP*) mice), gross tumors developed in a majority of the mice. Tumors arising in *SLAP* mice resembled human MCC tumors, both histologically and through transcriptomic comparison by RNA-seq analysis.

Of interest is the role that TP53 hemizygosity plays in the development of MCC in this mouse model. This is unexpected given that in human MCPyV+ MCC, the genetic sequence of TP53, and for pRB, is preserved [107,120]. However, their functions and downstream activity are impeded by the MCPyV T antigens [23,25,109]. The Dlugosz group hypothesizes that the necessity for using mice hemizygous for TP53 is due to differences in TP53-regulated genes between humans and mice. Another possibility is the species-restricted tumorigenic activity of MCPyV T antigens. It is possible that the ST antigen is unable to inactivate mouse TP53 activity as efficiently as it does human TP53 [25]. Nevertheless, this in vivo model is particularly exciting as this represents the first viable mouse model in which a murine MCC equivalent develops at a significant frequency, thus paving the way for future mechanistic studies focused on MCPyV T antigen-induced neoplastic progression.

## 6. Conclusions and Future Directions

In the context of MCPyV+ MCC, some of the most significant challenges in establishing biologically relevant in vitro and in vivo model systems relate to the lack of a well-defined natural target cell of MCPyV infection and MCC cell of origin. Additionally, the species specificity of human tumor-associated DNA viruses, including MCPyV, limits the ability to study the natural virus life cycle in laboratory animals. Despite these challenges, several relevant and valuable preclinical models for infection and disease have been established (Figure 2).

As the field progresses and novel scientific techniques are continuously established, future models for MCPyV replication and MCPyV+ MCC will likely better recapitulate the biology of the virus and associated disease. One such area of focus is elucidating the natural tropism of MCPyV, i.e., the cell into which MCPyV enters and completes its full viral life cycle. Better infection models can help illuminate cell fate post-infection and if the cell remains viable, which would be critical to addressing the origin of MCPyV+ MCC. In addition, the ubiquitous presence of MCPyV in other anatomical sites may indicate a tropism for a non-cutaneous cell type, and expanding the scope of future models will help test this hypothesis.

As more biological models are generated, well-known risk factors can be incorporated to investigate underlying mechanisms at play during the tumorigenesis process. In vivo models such as the *SLAP* mouse model described by Verhaegen et al. [137] are particularly exciting as it shows in vivo development of tumors that resemble human MCC tumors, thus potentially opening the avenue for incorporating risk factors such as UV radiation and immunosuppression [40]. Such studies would better characterize the interplay between these risk factors and MCPyV to help determine at which stage they are involved in the pathogenesis of MCC. Moreover, with the knowledge gained from in vivo Merkel cell development studies and associated signaling pathways [94,95,97,98,136,183], an exploration into the potential involvement of the MCPyV T antigens at various stages may also provide better insight into the possibility that T antigens utilize or bypass regulation of these pathways to drive MCC development. The information that can be gleaned from such studies would advance our collective knowledge in order to develop better strategies to treat such an aggressive disease that is predicted to become more prevalent in the near future [44].

The field of MCPyV and MCPyV+ MCC research continues to progress rapidly, and the in vitro and in vivo biological models described in this review are poised to become indispensable tools to further study MCPyV infection and MCC development. As the only polyomavirus thus far with a clear causative association with human malignancy, a greater understanding of MCPyV and its role in MCC will further our understanding of viral etiology in other diseases as well. MCC also presents as an interesting model for studying the immune responses to cancers for a few reasons: (i) different causative agents (MCPyV T antigens vs. UV-induced mutations); (ii) immune evasion of the virus and consequently the tumor itself. This may be through multiple mechanisms, allowing for the MCPyV+ MCC tumors to persist in spite of a heavy viral/neoantigen burden; (iii) the viral genes encoding the MCPyV T antigens are relatively small compared to other genes and, therefore, provides a technical advantage as a tool for immunological studies. For these reasons, further development and optimization of in vitro and in vivo models for future investigations of MCC, in general, have the potential to contribute to the understanding of the biology of cancer immunogenicity.
